# Outcomes of a structured inter-professional ultrasound guided vascular access workshop: a program evaluation report analyzing structure, participation and satisfaction

**DOI:** 10.15694/mep.2020.000002.1

**Published:** 2020-01-07

**Authors:** Taryn Jenna Rohringer, Rajat Chand, Simal Goman, Dimitri A. Parra

**Affiliations:** 1 University of Toronto; 2 John H. Stroger; 3 The Hospital for Sick Children

**Keywords:** Vascular access, ultrasound guidance, simulation based learning, deliberate practice, inter-professional workshop

## Abstract

This article was migrated. The article was marked as recommended.

Ultrasound-guided vascular access procedures are increasingly performed, particularly in the hospital setting, by a variety of health care professionals. Adequate teaching of the skills required for these procedures is important for all clinicians conducting these procedures. We created an inter-disciplinary workshop to teach these skills to anyone interested at our institution. This was a half-day workshop that combined pre-workshop teaching materials with didactic lectures and simulation based learning, which was followed by deliberate practice. Enrollment was on a first come first serve basis. We retrospectively reviewed the enrollment and performance of this workshop at our institution over 18 months. The workshop proved equally attractive to trainees and staff. Participants spanned a variety of healthcare disciplines, with the most common being intensive care (27%) and diagnostic imaging (23%). Participants indicated high satisfaction with the workshop, with a mean score of 4.7 on a 5-point Likert scale given to measure overall satisfaction. A long-term impact survey indicated regular use of skills learned in the workshop and a perceived improvement in clinical practice. This study helps demonstrate the efficacy of this inter-professional workshop structure in helping multidisciplinary healthcare professionals acquire unique skillsets for everyday clinical practice.

## Introduction

The ability to perform ultrasound-guided vascular access by a variety of medical and surgical disciplines is becoming an increasing need in highly complex hospitals. Indications are diverse and expanding, including nutrition, drug delivery, hydration and blood sampling ( Chait *et al.*, 2002; Westergaard, Classen and Walther-Larsen, 2013). Use of ultrasound guidance helps to reduce complications from vascular access procedures. Central venous line placement has a complication rate ranging from 5-26%, which include infection, pneumothorax, arterial puncture, deep vein thrombosis and bleeding ( Chait *et al.*, 2002; Westergaard, Classen and Walther-Larsen, 2013). These vascular access procedures are particularly challenging in the pediatric population due to factors such as small veins and poor visualization ( Westergaard, Classen and Walther-Larsen, 2013). Ultrasound guidance is therefore particularly useful in this population. Though image guidance has placed interventional radiologists at the forefront of performing these procedures, particularly in complex cases, other specialties such as emergency medicine physicians and critical care physicians commonly perform vascular access procedures ( Barsuk and Wayne, 2009; Barsuk *et al.*, 2009). Due to the increasing prevalence of ultrasound-guided vascular access procedures in the hospital setting and lack of standardized training in this skill across disciplines, a needs assessment was performed at our pediatric institution. This assessment concluded across a variety of specialties that a formal training opportunity should be provided to those interested in refining this important skill.

Studies have revealed that, compared to traditional clinical education, simulation-based learning (SBL) with deliberate practice (DP) is significantly more effective toward acquiring specific clinical skills ( McGaghie *et al.*, 2011). SBL put participants into lifelike experiences where they are required to use the skill being taught ( McGaghie *et al.*, 2011; Cohen *et al.*, 2013). DP involves a task with well-defined goals that learners are equipped to meet, and are motivated to focus on and repeatedly practice while receiving feedback and evaluation both during and following the practice ( McGaghie *et al.*, 2011). SBL has been demonstrated to be successful, specifically in helping learners acquire central venous catheter insertion skills ( Barsuk and Wayne, 2009; Barsuk *et al.*, 2009, 2010). SBL improved skills, decreased the number of attempted needle passes, and increased self-confidence for residents in an intensive care unit setting ( Barsuk and Wayne, 2009; Barsuk *et al.*, 2009). Moreover, learners exhibited high retention rates (82.4-87.1%) one-year following the SBL experience for central venous catheters ( Barsuk *et al.*, 2010).

The demonstrated efficacy of SBL interventions coupled with the results of our institution’s needs assessment resulted in the creation and implementation of an SBL workshop. This is a retrospective evaluation of the workshop, including its structure, participation, satisfaction and impact over 18 months.

## Description

This was a retrospective review of a structured, half-day ultrasound-guided vascular access workshop offered at The Hospital for Sick Children. Sessions were analyzed over an 18 month period. The maximum number of participants per session was 6. Sessions were advertised and made available to any interested health care professional that was affiliated with our institution. Enrollment occurred on a first come-first serve basis. Discipline and level of training of enrolled participants were recorded. An online teaching module and pre course reading material were delivered to the participants as a prerequisite. The module provided an interactive review of the basics of vascular access, including ultrasound orientation, aseptic technique, a step-by-step checklist of the procedure, and possible complications.

The workshop itself began with didactic lectures, covering ultrasound basics, the technique of ultrasound guided vascular access, types of central venous catheters, and finally an introduction to the use of the ultrasound machine. The didactic lectures were given by an interventional radiologist, ultrasound technologist, and vascular access nurses. Following this, participants were divided into groups of 2 and began the simulation based learning on task trainers (ultrasound compatible vascular access models). The groups rotated through three stations - a “Needle Guidance” station, a “Neck Jugular Access” station, and a “Baby Jugular and Femoral Access” station. Time was given for deliberate practice and final debriefing. Following the debrief, all participants completed a course evaluation. Participant satisfaction was measured using a validated Likert scale of 1 to 5. Parameters asked about included overall outcome, design, facilitator, and overall satisfaction. A long-term impact survey was delivered to participants 6-18 months after their participation in the course.

## Results

**Figure 1.**
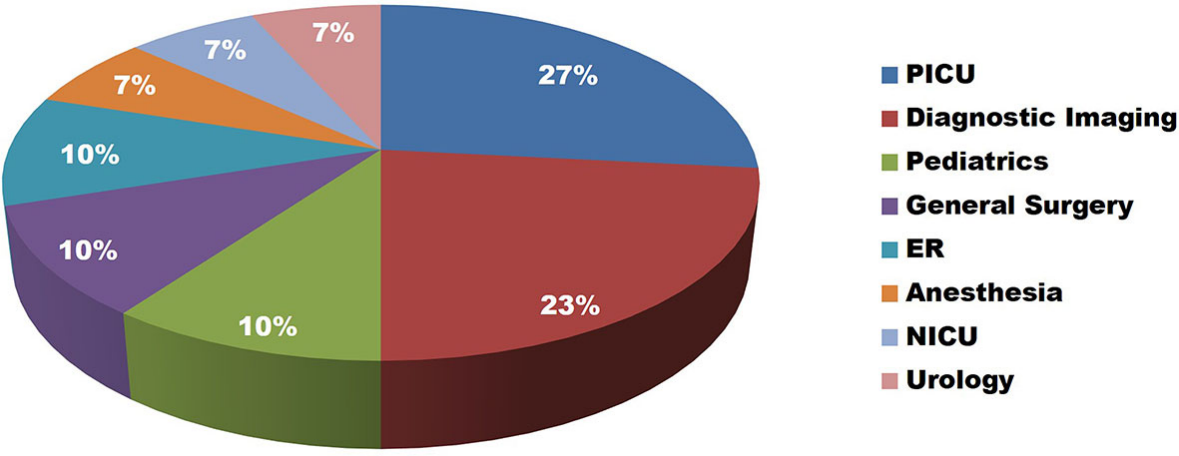
Pie graph illustrating the distribution of the specialties which participated in the workshop.

In the 18 month period reviewd, a total of 30 participants registered for the workshop. The majority of participants were from intensive care (27%) and diagnostic imaging (23%). Other disciplines represented in the workshop included pediatrics, general surgery, emergency medicine, and anesthesia ( Figure 1).

**Figure 2.**
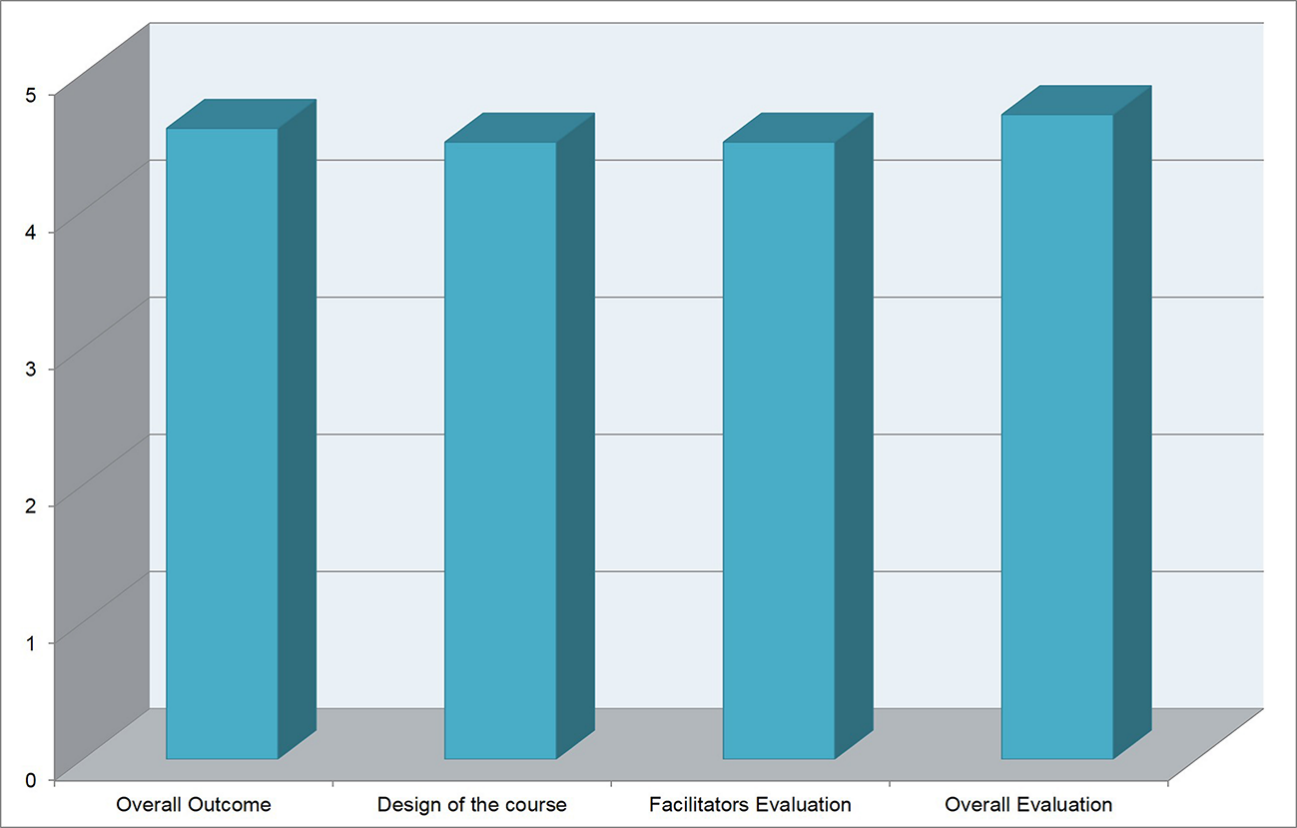
Graph showing the levels of satisfaction according to four different items.

Of the enrolled participants, 50% were staff and 50% were trainees. Based on the satisfaction survey, participants demonstrated high levels of satisfaction ( Figure 2).

**Figure 3.**
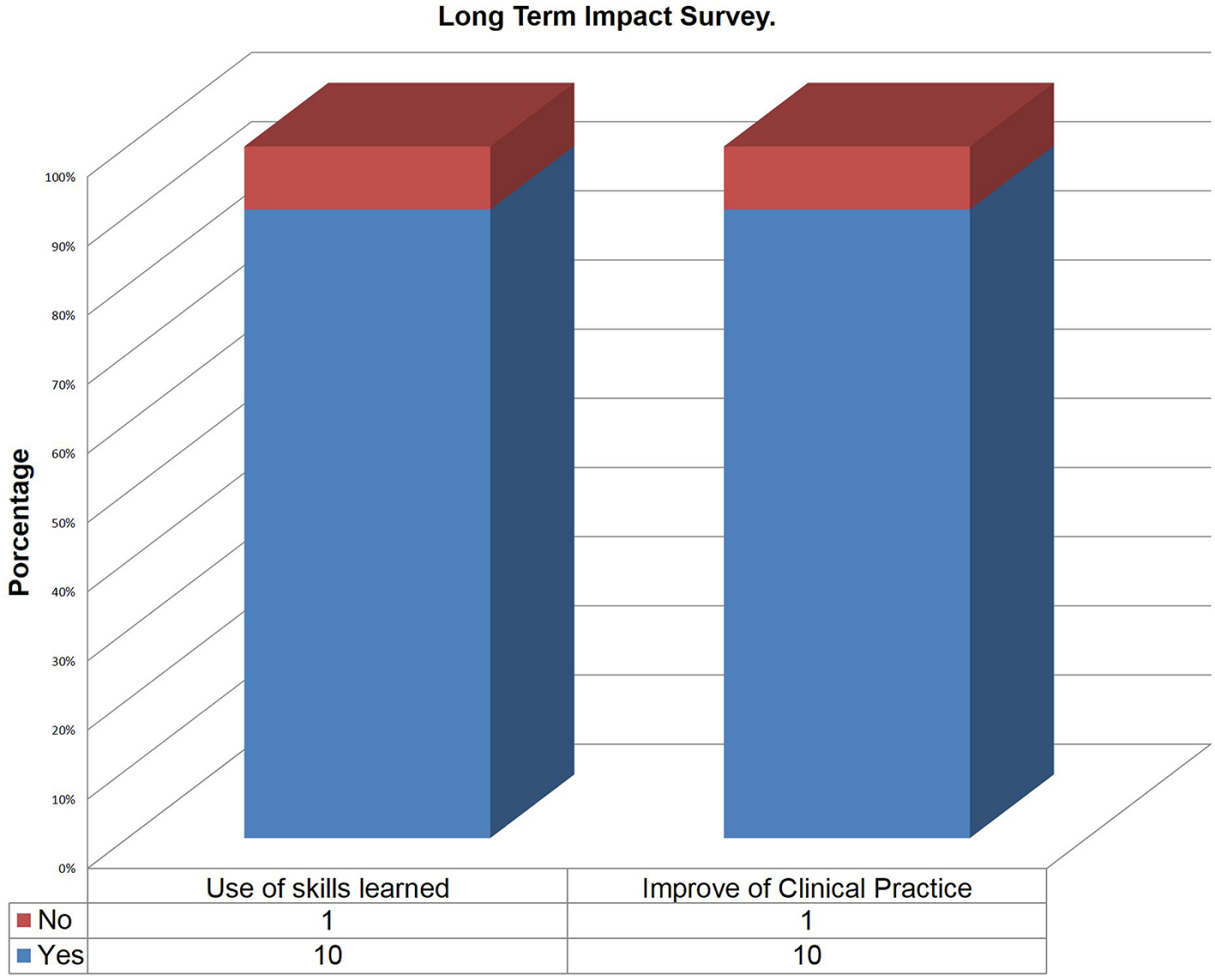
Graph illustrating the results of the long term impact survey. Eleven participants responded.

Mean score given for overall satisfaction was 4.7 on the 1-5 Likert scale. The long term impact survey was completed by 11 participants. Of the participants who responded, 90.9% indicated that they used the skills learned in the workshop and 90.9% indicated that their clinical practice improved as a result of the workshop ( Figure 3).

## Discussion

This retrospective review of our institution’s ultrasound-guided vascular access workshop demonstrates the success of this inter-professional and simulation based learning model. Central venous catheter insertion procedures are commonly performed. Advances in imaging techniques and employment in the procedural setting have increased accuracy and safety, leading to significantly fewer infections, associated costs, and line misplacements when compared to the traditional technique of surgically placed lines ( Chait *et al.*, 2002). Moreover, in children, these procedures, albeit potentially life-saving, can be very technically challenging and dangerous, owing to their small size ( Chait *et al.*, 2002; Westergaard, Classen and Walther-Larsen, 2013). Therefore, improved visualization using ultrasound is of particular pertinence ( Westergaard, Classen and Walther-Larsen, 2013).

With the increasing use of ultrasound guidance for central venous catheter placement, interventional radiologists are performing an increasing number of these procedures ( Chait *et al.*, 2002). However, central venous catheterization is commonly performed at the bedside in a variety of settings, such as the intensive care unit and the emergency department ( Barsuk and Wayne, 2009). Trainees and staff members from a variety of disciplines perform such procedures at the bedside, and as such require training on ultrasonic guidance and vascular access - a requirement that our institution concluded wasn’t optimally taught based on a needs assessment. This workshop effectively bridged this gap and was attractive to a diverse group of participants across multiple medical specialties ( Figure 1). It was also equally attractive to both trainees and staff. Moreover, the high satisfaction and long-term impact scores reinforce the success of a workshop for a multidisciplinary audience that is taught by a diverse set of health care professionals (i.e. trained technologists, nurses, physicians). We acknowledge that a limitation of this study is the subjective nature of the self-reported outcome measures of experience satisfaction and perceived skill improvement.

Furthermore, in addition to this workshop validating an interprofessional approach, it also helped to positively augment the literature on the success of SBL, which has been demonstrated in a variety of settings, including for teaching central venous catheterization skills ( Barsuk and Wayne, 2009; Barsuk *et al.*, 2009, 2010). In addition to pure SBL, our workshop’s structure of pre-experience modules and readings, multidisciplinary didactic lectures, SBL and deliberate practice, and debriefing helped lead to high participant satisfaction and long-term skill retention and clinical improvement ( Figures 2 and 3). This workshop is now offered 2-4 times per year at our institution.

## Conclusion

This study describes the success of an inter-professional ultrasound-guided vascular access workshop within our organization. We attribute such success to the multidisciplinary group of educators, mirroring the variety of specialties who registered for the course, as well as the combination of teaching methods, such as pre-workshop independent learning, didactic lectures, SBL, and deliberate practice. Participants had high levels of satisfaction with the workshop both immediately after and in a long-term impact survey.

## Take Home Messages

• Vascular access is increasingly being performed with ultrasound guidance by a number of different health care professionals who require this skill for daily practice.
• Ultrasound guided vascular access workshops appeal to trainees and staff in a variety of disciplines.
• An inter-professional workshop is effective in engaging and educating an inter-professional audience.
• Combining pre-workshop learning material, didactic lectures and simulation based learning with deliberate practice is an effective strategy.
• Our workshop led to participant satisfaction and long-term skill acquisition.

## Notes On Contributors

Taryn J. Rohringer is a medical student at the University of Toronto involved with a variety of research projects. She contributed to the data analysis of the study and she developed the manuscript. ORCID ID: https://orcid.org/0000-0002-8425-0036.

Dr Rajat Chand is a Radiology resident at John H. Stroger, Jr. Hospital of Cook County. He helped in the development of the course curriculum, e-learning module and contributed in the development of the manuscript.

Simal Goman is a research associate at the division of Image Guided Therapy at the Hospital for Sick Children. She contributed in the course design and data collection.

Dr Dimitri A. Parra is a Pediatric Interventional Radiologist at the Hospital for Sick Children and Assistant Professor at the University of Toronto. He developed, implemented and delivered the workshops, participated in the data analysis and in the development of the manuscript. ORCID ID: https://orcid.org/0000-0003-0214-3382.
