# Supplementary material for: Outcomes of a structured inter-professional ultrasound guided vascular access workshop: a program evaluation report analyzing structure, participation and satisfaction
Source: MedEdPublish (2016). 2020 Jan 7;9:2. [Version 1] doi: 10.15694/mep.2020.000002.1 (PMC10699378; doi:10.15694/mep.2020.000002.1)
Supplement: Peer review history

# Reviewer report: Reviewer response for version 1

**DOI:** 10.21956/mep.19972.r30363

**Published:** 2020-04-18

**Authors:** Ken Masters

**Affiliations:** 1 Sultan Qaboos University

This review has been migrated. The reviewer awarded 3 stars out of 5 An interesting brief report on the outcomes of a structured inter-professional ultrasound guided vascular access workshop. The authors begin well by clearly outlining the need for the skills to be taught in the workshop. I also liked the fact the workshops were restricted in number of participants. Although this does lessen the numerical impact of each workshop, it increases the ability of the individual participants to learn; repeating the workshop (as the authors have done) compensates for the small number of attendees in each run of the workshop. (It also lessens the burden on the departments who have to cope without their staff while they attend the workshop).Some points requiring addressing:• Although the authors do give a brief description of the workshop, there is little to go on (remember that part of the value of such a paper is that others may wish to implement the same). As a result, the value of the paper would be greatly enhanced if the authors could supply (as an Appendix or Supplementary File), a more detailed account of the workshop.• When numbers are given, it would be useful if raw numbers (followed by percentages in brackets) could be given, rather than percentages only. • Figure 2 is probably not necessary – the data could rather be expressed in a simple table. This would allow for a more accurate reading of the data (and also save space).• Similarly for Figure 3, especially as the data are given anyway. (And using the 3-d chart might look nicer, but is actually harder to read than a 2-d chart)I look forward to a Version 2 of this paper that addresses these points.

Reviewer Expertise:

NA

No decision status is available

# Reviewer report: Reviewer response for version 1

**DOI:** 10.21956/mep.19972.r30362

**Published:** 2020-01-08

**Authors:** Gert Van Zyl

**Affiliations:** 1 University of the Free State

This review has been migrated. The reviewer awarded 4 stars out of 5 A very important contribution to participate in the debate of improving skills. It is good to see results that indicated regular use of skills learned in the workshop and a perceived improvement in clinical practice. The number over 18 months might be small at this stage to determine a trend. Surprised that the number of attendees is equal between students and staff. Expected it to be higher for staff - but perhaps it was so in the beginning of the 18 months! I am of the opinion that the study needs to continue to try and determine a trend.

Reviewer Expertise:

NA

No decision status is available
